# Changes in oral function, swallowing function, and quality of life in patients with head and neck cancer: a prospective cohort study

**DOI:** 10.1186/s12903-022-02329-5

**Published:** 2022-07-17

**Authors:** Yoshiaki Ihara, Hirotaka Kato, Yuichi Tashimo, Yoshiki Iizumi, Yuma Fukunishi, Hitoshi Sato, Toshikazu Shimane, Koji Takahashi

**Affiliations:** 1 Division of Oral Functional Rehabilitation Medicine, Department of Special Needs Dentistry, School of Dentistry, Showa University, Kitasenzoku 2-1-1, Oh-taku, Tokyo, 145-8515 Japan; 2 Head and Neck Oncology Center, Showa University Hospital, Tokyo, Japan

**Keywords:** Head and neck cancer, Dysphagia, Oral function, Quality of life, Morbidity, Swallowing

## Abstract

**Background:**

Head and neck cancer (HNC) treatment can cause oral morbidities, such as oral dryness and dysphagia, affecting the patient’s quality of life (QOL). The relationship between oral functions and QOL in patients with early-stage HNC remains poorly studied. This study aimed to evaluate changes in the QOL of patients with early-stage HNC and identify factors that affect the QOL of these patients.

**Methods:**

In this prospective cohort study, 37 patients who underwent early-stage (Stage I/Stage II) HNC treatment were evaluated for their oral function, swallowing function, and the QOL score at baseline (BL) and 12 months after surgical treatment (12 M). The participants were divided into two groups: patients who returned to the BL QOL score at 12 M (RE; n = 26) and those who did not (NR; n = 11).

**Results:**

In total, 29.7% (11/37) patients with early-stage HNC did not return to the BL QOL score at 12 M. There was no significant difference between the RE and NR groups regarding the oral and swallowing function. Moreover, oral and swallowing function of all patients returned to the BL at 12 M. The NR group showed lower QOL scores than the RE group in the global health status, and “sticky saliva” parameters in the questionnaires.

**Conclusion:**

Restoration of the oral function is insufficient to improve the QOL of patients with early-stage HNC. The treatment of these patients should instead consider several factors that affect their QOL.

## Background

Based on anatomy and topography, head and neck cancer (HNC) defines malignant upper aerodigestive tract tumors, including those of the oral cavity, pharynx, and larynx. HNC is the ninth most common malignant neoplasm in the world [1]. HNC treatments affect not only the patients’ oral functions such as swallowing and speech but also their cosmetic and psychological function [2]. The acute side effects associated with HNC treatment may persist immediately following treatment, while the chronic side effects may develop after ≥ 90 days [3, 4].

Common oral morbidities associated with HNC treatment include difficulty in swallowing (dysphagia), oral dryness (xerostomia), difficulty in mouth opening (trismus), oral pain, and taste and smell alterations [5]. Dysphagia is one of the most common symptoms resulting from HNC treatment [4, 6], which may be caused by surgery, external-beam radiotherapy, and concurrent chemotherapy for HNC. It has been reported in 50–70% of patients with HNC and may be a consequence of both acute and chronic complications associated with HNC treatment [7–9]. Other oral morbidities associated with HNC treatment that affect the oral function of patients with HNC include xerostomia and trismus [8, 10]. These oral morbidities also decrease the patients’ quality of life (QOL) [11, 12].

The prevalence of HNC among younger individuals and survival rates of patients with HNC are increasing [13, 14]. Evaluating the QOL among patients with cancer is important to understand the impact of the disease and its treatment on the patient’s routine life and improve the care protocol with more comprehensive clinical and rehabilitation support measures [15]. Therefore, maintaining both oral function and QOL is important for patients with HNC, and HNC treatment should be considered based on the patient’s post-treatment functional outcomes and QOL. The QOL of patients with HNC reportedly returns to the baseline (pre-treatment) after 12–18 months post-treatment [16, 17].

The tumor stage is reportedly associated with the patient’s QOL after treatment [18]. The treatment of early-stage HNC might have small effects on a patient’s QOL [18, 19]. However, some patients complain of discomfort in the oral function a year after completing HNC treatment. Therefore, we hypothesized that there are other influences such as patient’s social condition and psychological background on patients' QOL besides oral function. This study aimed to evaluate changes in the QOL of a patient with early-stage HNC and to identify factors that affect the QOL of these patients following HNC treatment.

## Methods

### Patients

This prospective cohort study included 37 patients with early-stage HNC (Stage I or Stage II). All patients were received for HNC treatment at the Head and Neck Oncology Center of our hospital, and were referred to our department for rehabilitation of swallowing and/or speech problem. The exclusion criteria comprised (1) patients aged under 20 years, (2) patients who could not follow our instructions of the assessments, (3) patients who had other tumors, (4) patients who had severe systemic diseases that may had effect on the swallowing function, and (5) patients who did not complete measurement data. The assessments were evaluated before treatment (baseline: BL) and 12 months after treatment (12 M).

Based on The European Organization for Research and Treatment of Cancer (EORTC) QLQ-C30 (global health status), participants were divided into two groups: patients who returned to the BL QOL score at 12 M (RE) and those who did not return to the BL QOL score at 12 M (NR).

This study was approved by the Ethics Committee of our university (Approval no. 2355). Before participating in this study, all patients received both oral and written informed consent, and signed an approved written informed consent form. This study was conducted in accordance with the World Medical Association Declaration of Helsinki (version 2002).

### Assessments

Assessments were undertaken by the dentists of our department. The data pertaining to the primary tumor site, the TNM classification, method of the HNC treatment, and medical history of each patient were collected from patients’ medical records. The oral function measurements included Lip closure pressure (LC), tongue pressure (TP), and oral moisture (OM) Swallowing function measurements included the Mann Assessment of Swallowing Ability–Cancer version (MASA-C) and the Functional Oral Intake Scale (FOIS). The EORTC QLQ-C30 and QLQ-H&N35 questionnaires Japanese version were used for evaluating QOL of patients.

### Oral function measurements

#### LC

An LC pressure measuring device “Lip de Cum” (LDC-110R Lip De Cum lip force measurement device; Ducklings, Cosmo Instruments Co., Ltd., Tokyo, Japan) was used to evaluate LC. Patients were instructed to close their lips as hard as they could. Moreover, a maximum LC force was recorded. Five measurements were taken, and the mean score of the all measurements was calculated and used as the patient’s LC score [20].

#### TP

A JMS tongue pressure measuring device (TPM-01, JMS Co. Ltd., Hiroshima, Japan) was used to evaluate TP. An intraoral balloon-shaped probe was placed at the center of the tongue, positioned behind the upper incisors of the hard palate. During all measurements the patients were instructed to close their lips, and press their tongues against their hard palates to push the probe using maximum tongue force. The maximum air pressure of the probe was then recorded on the device. Measurements were taken 10 times, and the mean score of all the measurements was calculated and used as the patient’s TP score. Each measurement was taken with an interval of at least 1 min between the measurements [20].

#### OM

An oral moisture-checking device “Mucus®” (Moisture Checker for Mucus®, Life Co. Ltd, Tokyo, Japan), which could measure the moisture content in the oral mucosa indirectly based on the capacitance method [21] was used to evaluate OM. Five times measurements were undertaken. The mean score of the all measurements was calculated and used as the patient’s OM score [20, 22].

### Swallowing function

#### MASA-C

The MASA-C that has been validated for use in patients with HNC to identify dysphagia was used to evaluate patient’s swallowing function. The total maximum score of MASA-C is 200 points. In this study, a cut-off score to identify dysphagia was 185 [23].

#### FOIS

The functional eating status was evaluated using the FOIS. The FOIS is a reliable and valid 7-point ordinal scale to assess functional oral intake of materials in patients with oropharyngeal dysphagia [24].

### QOL measurements

The patient’s QOL was evaluated using the EORTC QLQ-C30 version 3.0 and QLQ-H&N35 questionnaires Japanese version [25, 26]. According to the EORCTC scoring manual, the scores of patients QOL were calculated [27, 28]. The EORTC QLQ-C30 and EORTC H&N35 have been applied in a study on the QOL of laryngeal cancer patients treated with radiotherapy [29]. The scores of the QLQ-C30 and QLQ-H&N35 items were linearly transformed to a scale of 0 to 100. For functioning scales and global QOL scales, higher scores correspond to better levels of functioning. Conversely, for symptom scales, higher scores represent higher levels of symptoms or problems.

### Rehabilitation

If necessary, direct and/or indirect training including massage for surgical scarring, oral motor exercises such as tongue exercise was provided to all patients participating in this study. Rehabilitation was performed by dentists and speech-language pathologist. Moreover, the patients also underwent speech therapy by speech-language pathologists according to their requirements.

### Statistical analysis

The differences in oral functions and swallowing function between BL and 12 M were analyzed using the repeated measures analysis of variance. The differences in FOIS and QOL measurements were analyzed using Wilcoxon signed-rank test. The differences between RE and NR were analyzed using Wilcoxon signed-rank test. A *p* < 0.05 was deemed statistically significant.

## Results

### Patients

We included 37 patients (17 men; 20 women). The mean patient age was 62.67 years (standard deviation [SD]: 13.50 years). The participants were divided into RE (n = 26) and NR (n = 11) groups. The patients’ characteristics (age, sex, TMN classification, primary tumor site) are described in detail in Table 1. In the RE group, 23 patients (88.5%) underwent surgical treatment (partial glosseectomy; 11, partial laryngopharyngectomy; four, other; nine), one (3.8%) underwent chemoradiotherapy (CRT) (Cisplatin, 70 Gy), and two (7.7%) underwent radiotherapy (RT) (av. 68 Gy). In the NR group, eight patients (72.7%) underwent surgical treatment (partial glosseectomy; five, partial laryngopharyngectomy; three), one (9.1%) underwent CRT (Cisplatin, 70 Gy), and two (18.2%) underwent RT (av. 65 Gy).

**Table 1:** Patient demographics

Variable	RE	NR
Age (mean and SD)	62.33 (15.18)	63.42 (9.17)
Sex (male:female)	11:15	6:5
Primary tumor site		
Nasopharynx	5	1
Faucial arch	1	1
Tongue	11	5
Hypopharynx	4	4
Other	5	0
Tumor stage	Number (%)	Number (%)
T1N0 (stageI)	13 (50.0)	9 (81.8)
T2N0 (stage II)	13 (50.0)	2 (18.2)

### Oral function measurements

#### LC

**Fig. 1:**
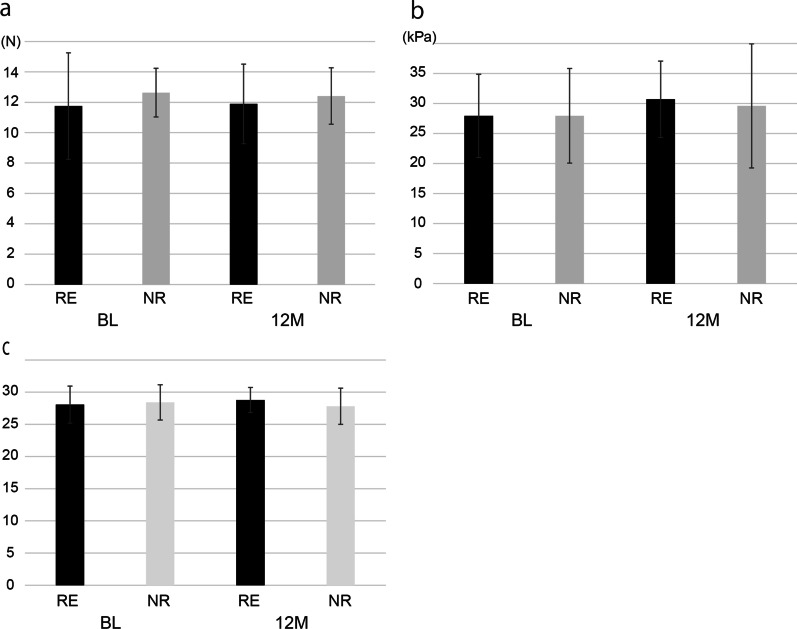
The results of oral functions. **a** The results of LC. There was no significant difference about the mean LC between RE and NR at both BL and 12 M. **b** The results of TP. There was no significant difference about the mean TP between RE and NR at both BL and 12 M. **c** The results of OM. There was no significant difference about the mean OM between RE and NR at both BL and 12 M

At BL, the mean LC in the RE and NR group was 11.75 N (SD = 3.50) and 12.63 N (SD = 1.60), respectively, whereas, at 12 M, the mean LC was 11.89 N (SD = 2.62) and 12.41 N (SD = 1.86), respectively (Fig. 1a). There was no significant difference between the groups at both timepoints (BL: *p* = 0.41, 12 M: *p* = 0.56). Moreover, there were no significant differences between the mean LC at BL and that at 12 M in both the RE and NR groups (RE: *p* = 0.14, NR: *p* = 0.37).

#### TP

At BL, the mean TP in the RE and NR group was 27.94 kPa (SD = 6.94) and 27.95 kPa (SD = 7.89), respectively, whereas at 12 M, the mean TP was 30.69 kPa (SD = 6.36) and 29.60 kPa (SD = 10.34), respectively (Fig. 1b). There was no significant difference between the groups at both timepoints (BL: *p* = 0.94, 12 M: *p* = 0.87). Moreover, there were no significant differences between the mean TP at BL and that at 12 M in both the RE and NR groups (RE: *p* = 0.52, NR: *p* = 0.41).

#### OM

At BL, the mean OM in the RE and NR group was 28.07 (SD = 2.87) and 28.41 (SD = 2.74), respectively, whereas at 12 M, the mean OM was 28.77 (SD = 1.96) and 27.81 (SD = 2.81), respectively (Fig. 1c). There was no significant difference between the groups at both timepoints (BL: *p* = 0.73, 12 M: *p* = 0.24). Moreover, there were no significant differences between the mean OM score at BL and that at 12 M in both the RE and NR groups (RE: *p* = 0.49, NR: *p* = 0.31).

### Swallowing function measurements

#### MASA-C

**Fig. 2:**
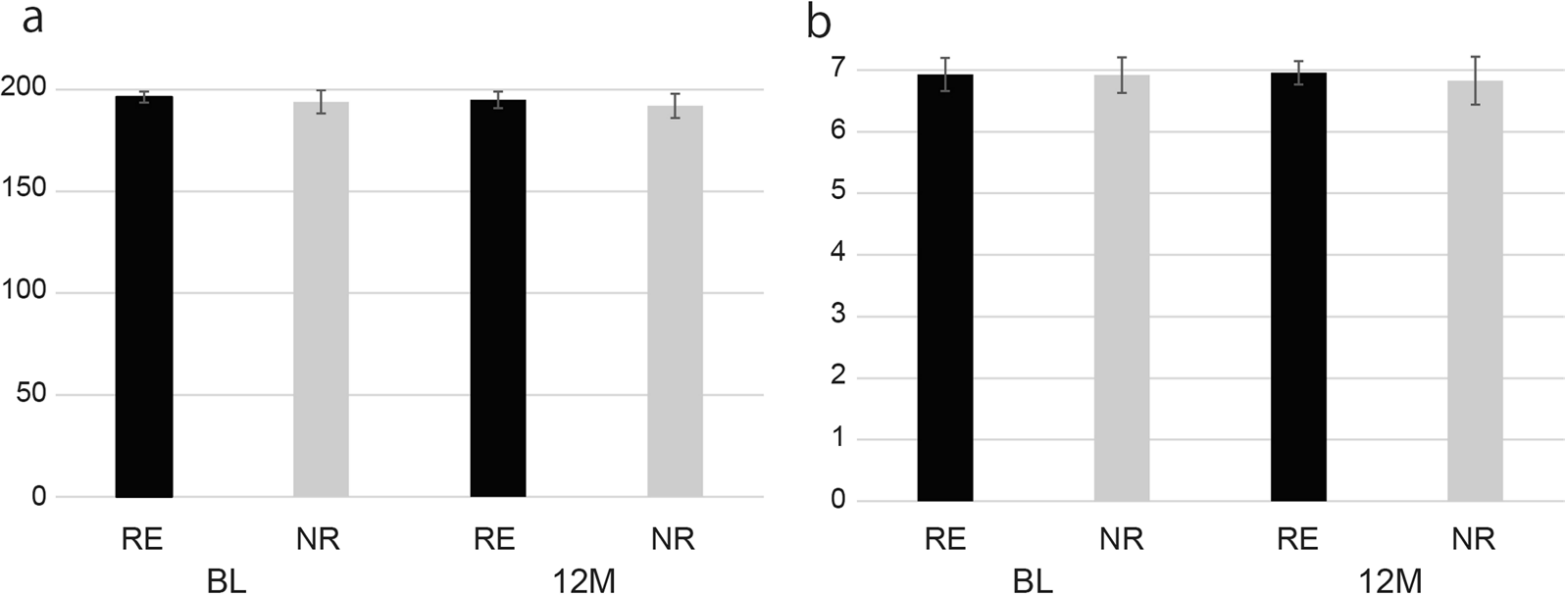
The results of swallowing functions **a**: The results of MASA-C. There was no significant difference about the mean MASA-C score between RE and NR at both BL and 12 M.** b**: The results of FOIS. There was no significant difference about the mean FOIS score between RE and NR at both BL and 12 M

At BL, the mean MASA-C score in the RE and NR group was 196.33 (SD = 2.66) and 194.00 (SD = 5.70), respectively, whereas at 12 M, the mean MASA-C score was 194.95 (SD = 4.08) and 192.00 (SD = 5.89), respectively. There was no significant difference between the groups at both BL (*p* = 0.29) and 12 M (*p* = 0.31) (Fig. 2a). At BL, all patients reported experiencing dysphagia (MASA-C score < 185), whereas at 12 M, only one patient in the NR group reported experiencing dysphagia (MASA-C score = 182). There were no significant differences between the mean MASA-C score at BL and that at 12 M in both the RE and NR groups (RE: *p* = 0.19, NR: *p* = 0.33) (Fig. 2a).

#### FOIS

At BL, the mean FOIS score in the RE and NR group was 6.93 (SD = 0.27) and 6.92 (SD = 0.29), respectively, whereas at 12 M, the mean FOIS score was 6.96 (SD = 0.19) and 6.83 (SD = 0.39), respectively. There was no significant difference between the groups at both timepoints (BL: *p* = 0.93, 12 M: *p* = 0.29) (Fig. 2b). Moreover, there were no significant differences between the mean FOIS score at BL and that at 12 M in both the RE and NR groups (RE: *p* = 0.62, NR: *p* = 0.11) (Fig. 2b).

### QOL measurements

Tables 2 and 3 present the results of the EORTC QLQ-C30 and QLQ-H&N35 questionnaires. At BL, no significant differences were noted in both the EORTC QLQ-C30 and QLQ-H&N35 questionnaire scores between the RE and NR groups, whereas at 12 M, the global health status in the EORTC QLQ-C30 for the RE group were significantly higher than those for the NR group (global health status; RE, 85.49 [SD = 12.99]; NR, 63.89 [SD = 13.45]; *p* < 0.01. In terms of global health status, five patients reported ‘a little’ change (5–10), five reported ‘moderate’ change (10–20), and two reported ‘very much’ change (> 20) in the NR group [26]. In the EORTC QLQ-H&N35, only the “sticky saliva” score in the RE group was significantly lower than that in the NR group (RE, 12.35 [SD = 4.54], NR, 25.00 [SD = 20.72], *p* = 0.02).

**Table 2:** Result of EORTC QLQ-C30

		BL		12 M	
		Mean	95% CI^a^	Mean	95% CI^a^
Global health status	RE	70.35	(64.396; 76.30)	85.49	(80.35; 90.63)
	NR	79.08	(70.73; 87.43)	63.89	(55.34; 72.44)*
Physical functioning	RE	96.91	(93.88; 99.95)	94.07	(90.05; 98.10)
	NR	97.78	(95.02; 100.54)	88.33	(78.93; 97.75)
Role functioning	RE	94.44	(89.97; 98.92)	96.30	(92.49; 100.10)
	NR	100	(100; 100)	93.06	(84.66; 101.45)
Emotional functioning	RE	83.95	(78.10; 89.80)	90.43	(86.27; 94.59)
	NR	90.97	(81.27; 89.80)	95.14	(89.86; 100.41)
Cognitive functioning	RE	85.80	(80.11; 91.50)	85.80	(79.28; 92.32)
	NR	91.67	(84.53; 98.81)	84.72	(75.19; 94.26)
Social functioning	RE	90.74	(86.14; 95.34)	96.30	(92.07; 100.52)
	NR	86.11	(70.58; 101.64)	98.61	(95.55; 101.67)
Fatigue	RE	18.52	(13.64; 23.39)	16.05	(10.97; 21.12)
	NR	10.19	(2.53; 17.84)	17.73	(10.96; 33.49)
Nausea and vomiting	RE	1.85	(− 0.94; 4.65)	4.32	(− 1.34; 9.98)
	NR	2.78	(− 3.34; 8.89)	1.39	(− 1.67; 4.45)
Pain	RE	16.67	(8.91; 24.42)	6.79	(1.53; 12.05)
	NR	8.33	(1.19; 15.47)	15.28	(1.39; 29.16)
Dyspnea	RE	6.17	(− 0.20; 12.55)	2.47	(− 1.05; 5.99)
	NR	2.78	(− 3.34; 8.89)	5.56	(− 2.69; 13.80)
Sleep	RE	14.81	(6.37; 23.26)	8.64	(1.71; 15.57)
	NR	8.33	(− 1.25; 17.91)	11.11	(0.68; 21.54)
Appetite loss	RE	8.64	(2.75; 14.53)	9.88	(2.73; 17.02)
	NR	2.78	(− 3.34; 8.89)	5.56	(− 2.69; 13.80)
Constipation	RE	11.11	(2.93; 19.29)	6.17	(0.95; 11.39)
	NR	11.11	(− 5.38; 27.60)	8.33	(− 1.25; 17.91)
Diarrhea	RE	11.11	(4.78; 17.44)	6.17	(0.95; 11.39)
	NR	8.33	(− 1.25; 17.91)	8.33	(− 1.25; 17.91)
Financial difficulties	RE	11.11	(0.77; 21.46)	4.94	(− 1.08; 10.95)
	NR	8.33	(− 10.01; 26.67)	0	(0; 0)

EORTC, European Organization for Research and Treatment of Cancer; BL, baseline; 12 M, 12 months after treatment; RE, patients who returned QOL score to the BL at 12 M; NR, patients who did not return QOL score to the BL at 12 M

*p < 0.05, **p < 0.01

^a^Wilcoxon signed-rank test

**Table 3:** Result of EORTC QLQ-H&N35

		BL		12 M	
		Mean	95% CI^a^	Mean	95% CI^a^
Pain	RE	10.49	(7.97; 13.01)	4.81	(1.10; 8.52)
	NR	7.64	(3.44; 11.84)	8.33	(1.95; 14.72)
Swallowing	RE	10.49	(3.03; 17.95)	9.62	(4.17; 15.06)
	NR	6.94	(1.98; 11.91)	9.03	(2.46; 15.59)
Senses problems	RE	1.23	(− 0.53; 2.99)	5.13	(0.55; 9.70)
	NR	2.78	(− 1.34; 6.90)	5.56	(− 1.34; 12.45)
Speech problems	RE	7.41	(1.56; 13.25)	9.88	(5.26; 14.49)
	NR	8.33	(− 0.25; 16.91)	10.19	(4.59; 15.78)
Trouble with social eating	RE	16.67	(12.79; 20.54)	13.89	(8.40; 19.37)
	NR	9.72	(3.40; 16.04)	11.32	(8.55; 22.93)
Trouble with social contact	RE	2.47	(0.14; 4.80)	4.44	(0.94; 7.95)
	NR	6.67	(− 3.06; 16.39)	5.14	(− 2.41; 12.69)
Less sexuality	RE	27.78	(14.72; 40.84)	10.67	(1.17; 20.17)
	NR	15.15	(− 0.25; 30.55)	15.15	(− 0.25; 30.55)
Teeth	RE	11.11	(2.15; 20.07)	2.47	(− 1.05; 5.99)
	NR	5.56	(− 2.69; 13.80)	5.56	(− 2.69; 13.80)
Opening mouth	RE	11.11	(4.78; 17.45)	2.47	(− 1.05; 5.99)
	NR	8.33	(− 1.25; 17.91)	0	(0; 0)
Dry mouth	RE	18.52	(8.61; 28.42)	24.69	(16.04; 33.34)
	NR	11.11	(0.68; 21.54)	25.00	(2.65; 47.35)
Sticky saliva	RE	9.88	(3.74; 16.01)	12.35	(2.57; 22.12)
	NR	16.67	(5.61; 27.73)	25.00	(11.84; 38.16)*
Coughing	RE	16.05	(4.85; 27.24)	11.11	(4.78; 17.45)
	NR	11.11	(0.68; 21.54)	17.16	(8.54; 30.35)
Felt ill	RE	22.22	(11.88; 32.57)	11.11	(3.80; 18.43)
	NR	11.11	(0.68; 21.54)	22.22	(3.42; 41.02)
Pain killers	RE	7.40	(1.82; 12.99)	3.70	(− 0.52; 7.93)
	NR	5.56	(− 2.69; 13.80)	0	(0; 0)
Nutritional supplements	RE	1.23	(− 1.30; 3.77)	3.70	(− 0.52; 7.93)
	NR	2.78	(− 3.34; 8.89)	5.56	(− 2.69; 13.80)
Feeding tube	RE	1.23	(− 1.30; 3.77)	1.23	(− 1.30; 3.77)
	NR	0	(0; 0)	0	(0; 0)
Weight loss	RE	2.47	(− 1.05; 5.99)	8.64	(2.75; 14.53)
	NR	2.78	(− 3.34; 8.89)	5.56	(− 2.69; 13.80)
Weight gain	RE	6.17	(0.95; 11.39)	9.88	(3.74; 16.01)
	NR	8.33	(− 1.25; 17.91)	8.33	(− .25; 17.91)

EORTC, European Organization for Research and Treatment of Cancer; BL, baseline; 12 M, 12 months after treatment; RE, patients who returned QOL score to the BL at 12 M; NR, patients who did not return QOL score to the BL at 12 M

*p < 0.05, **p < 0.01

^a^Wilcoxon signed-rank test

## Discussion

In this study, we observed that oral and swallowing functions returned at 12 months following treatment in patients with early-stage HNC. A total of 29.7% (11/37) patients with an early-stage HNC did not return to the BL QOL score at 12 M. The patients who did not return to the BL QOL score at 12 M indicated a lower QOL than those who returned to BL QOL score at 12 M based on the global health status in the EORTC QLQ-C30 and the “sticky saliva” score in the QLQ-H&N35.

The oral functions in both the RE and NR groups returned to the BL at 12 M in the present study. Among the total patients included, 43.2% (16/37) were those with tongue cancer. The TP in both the RE and NR groups returned to the BL at 12 M. The TP values of patients with tongue cancer decrease following treatment, even in cases managed by minimal glossectomy [30]. The patients enrolled in this study received indirect and/or direct training such as jaw-opening exercises [31], tongue-to-palate pressure generation, and tongue muscle exercises. These rehabilitations might improve the patient’s TP.

A previous study reported that LC in patients with HNC returned to the BL 3 months following treatment [32]. The primary tumor site in their study included the tongue, pharynx, or maxilla. The patients enrolled in the present study included the same primary tumor sites. Therefore, the same tendency as that observed in the previous study might have been observed in our study at 12 M. Moreover, no patients with lip cancer were enrolled in this study. Therefore, no significant difference was observed between the mean LC score at BL and that at 12 M in both the RE and NR groups. The patients included in this study received rehabilitation as needed. Post-treatment rehabilitation affects the patient's QOL [33], and in this study, it was thought to have affected not only the results of function measurements but also results of QOL measurements.

Further, the swallowing function and eating ability in the patients with early-stage HNC returned at 12 M in our study. Based on the MASA-C scores, only one patient (1/52, 1.9%) reported experiencing dysphagia at 12 M. A previous study that focused on patients with early-stage HNC reported excellent swallowing outcomes, assessed using the MD Anderson Dysphagia Inventory (MDADI), within 1 year after treatment [34]. Their study evaluated patient’s QOL using the MDADI questionnaire. Both the present study and their study evaluated dysphagia using assessment tools. However, some studies evaluated dysphagia using videofluoroscopic or video-endoscopic examinations of swallowing [17, 35]. In the present study, we did not evaluate the patients’ swallowing function in terms of penetration and aspiration using videofluoroscopic or video-endoscopic examinations of swallowing. It has been previously reported that 57% of patients with HNC experienced aspiration during fiber-optic endoscopic swallowing evaluation [36]. Penetration and aspiration have been used as the main indicators of dysphagia. However, penetration and aspiration are not necessarily the same as dysphagia. Thus, it is necessary to evaluate the patient’s swallowing function based on both dysphagia using assessment tools, such as MASA-C and MDADI and penetration and aspiration using instrumental assessment, such as videofluoroscopic or video-endoscopic examinations of swallowing.

In a previous report, the swallowing and speech function and QOL-associated parameters in patients with early-stage HNC revealed excellent results [34]. The results of the present study showed the same tendency. The oral and swallowing functions and the swallowing function (swallowing, trouble with social eating, opening mouth, and dry mouth in the EORTC QLQ-H&N 35) and speech function (Speech problems and trouble with social eating form EORTC QLQ-H&N 35) parameters in the QOL assessment returned to the BL at 12 M. Another study has reported that the patients’ QOL with T1 and T2 tumor showed good QOL improvement with low symptom scores [16]. That study differs from ours in that it evaluated only early glottic carcinoma and treated it with transoral CO_2_ laser microsurgery (TLM), whereas in our study, the patients enrolled had several primary5 tumor sites and the treatment methods included CRT, RT, and surgical treatment. Moreover, Hendriksma et al. included only T1N0 and T2N0 patients due to TLM indications. Differences in treatment methods (dose of RT, chemotherapy regimens) have different effects on a patient’s QOL [37]. The use of RT is a major determinant of the QOL of patients with cancer [38]. Treatment with primary surgery or primary radiation in patients with HNC has a strong prognostic association with their QOL [39]. Moreover, the addition of chemotherapy to curative radiation indicates a trend toward a worse QOL [40]. The difference in the treatment methods for HNC indicates different morbidities in the swallowing function [41]. Unlike external-beam RT and CRT, surgical treatment has less additional chronic adverse effects on oral function [42]. Furthermore, the health-related QOL of patients with squamous cell carcinoma is not influenced by tumor location [43]. Thus, the difference in the treatment methods is a possible reason for decreased QOL in 29.7% of patients (11/37) at 12 M in the present study.

Our study indicated no significant differences in OM between the RE and NR groups. However, there was a significant difference in the “sticky saliva” score in the QLQ-H&N35 questionnaire between the RE and NR groups. Aging has been reported to affect the salivary glands, salivary flow rate, and quality of saliva [44]. HNC treatment causes xerostomia [45]. However, there were no significant differences in age, sex, and treatment methods between the RE and NR groups in this study. OM and the amount of saliva have been reported to be associated with oral dryness [22]. However, there may not necessarily be a relationship between OM and the properties of saliva [46]. This could explain the present study results, which found no significant difference in OM between the RE and NR groups; however, there might have been differences in the properties of saliva between the groups. Salivary viscosity might have been higher in the NR group than in the RE group, which might have decreased the “sticky saliva” score in the latter group.

### Limitation

This study was a prospective cohort study with a small sample size because of the reduction in analyzed cases as a result of participant withdrawal during the study. Patient drop-out during a prospective HNC study is not unusual [47]. Moreover, it was a single-center study, which relied upon the expertise of the treating clinicians. Further, additional variables, such as saliva properties, the type, dosage, and duration of medications (for example; pain management medications, oral moisturizer), might provide insight into the results of the patients’ QOL. Moreover, the cases in this study included patients with several primary tumor sites who underwent several treatment methods for HNC. Therefore, future studies incorporating large number of patients and assessment of additional variables that might have influenced the outcomes of the present study are warranted. In this study, rehabilitation after HNC treatment was provided to all the patients as needed. Therefore, it is necessary to evaluate the effects of rehabilitation after HNC treatment on patients QOL.

## Conclusion

The oral and swallowing functions in patients with early-stage HNC returned to BL at 12 M. However, some patients did not return to their BL QOL score after treatment. These results suggested that restoration of the oral function is insufficient to improve the QOL in patients with early-stage HNC. Therefore, it is necessary to treat these patients with consideration of several factors such as patient’s social and phycological condition that affect their QOL.

## Data Availability

The datasets during the current study are available from the corresponding author on reasonable request.

## Abbreviations

HNC: Head and neck cancer
QOL: Quality of life
EORTC: The European Organization for Research and Treatment of Cancer
LC: Lip closure force
TP: Tongue pressure
OM: Oral moisture
MASA-C: The Mann Assessment of Swallowing Ability–Cancer Version
MDADI: MD Anderson Dysphagia Inventory
FOIS: Functional Oral Intake Scale
RE: Patients who returned to the baseline QOL Score at 12 M
NR: Patients who did not return to the baseline QOL Score at 12 M
